# The respiratory chain of *Klebsiella aerogenes* in urine-like conditions: critical roles of NDH-2 and *bd*-terminal oxidases

**DOI:** 10.3389/fmicb.2024.1479714

**Published:** 2024-11-06

**Authors:** Martín A. González-Montalvo, Jennifer M. Sorescu, Gabriella Baltes, Oscar Juárez, Karina Tuz

**Affiliations:** Department of Biological Sciences, Illinois Institute of Technology, Chicago, IL, United States

**Keywords:** bacteria metabolism, *bd*-terminal oxidase, *Enterobacter aerogenes*, *Klebsiella aerogenes*, NDH-2, oxidase, urine

## Abstract

*Klebsiella aerogenes* is an opportunistic nosocomial bacterial pathogen that commonly causes urinary tract infections. Over the past decades, *K. aerogenes* strains have acquired resistance to common antibiotics that has led to the rise of multidrug-resistant and even pandrug-resistant strains. Infections produced by these strains are nearly impossible to treat, which makes *K. aerogenes* a global priority to develop new antibiotics and there is an urgent need to identify targets to treat infections against this pathogen. However, very little is known about the metabolism and metabolic adaptations of this bacterium in infection sites. In this work, we investigated the respiratory metabolism of *K. aerogenes* in conditions that resemble human urine, allowing us to identify novel targets for antibiotic development. Here we describe that, unlike other gram-negative pathogens, *K. aerogenes* utilizes the type-2 NADH dehydrogenase (NDH-2) as the main entry point for electrons in the respiratory chain in all growth conditions evaluated. Additionally, in urine-like media, the aerobic metabolism as a whole is upregulated, with significant increases in succinate and lactate dehydrogenase activity. Moreover, our data show that the *bd*-I type oxidoreductases are the main terminal oxidases of this microorganism. Our findings support an initial identification of NDH-2 and *bd*-I oxidase as attractive targets for the development of new drugs against *K. aerogenes* as they are not found in human hosts.

## Introduction

*Klebsiella aerogenes*, formerly *Enterobacter aerogenes*, is a gram-negative, rod-shaped, motile, non-spore-forming bacterium (Grimont and Grimont, 2005). *K. aerogenes* is found in a plethora of niches, ranging from soil and water to the human and animal microbiota, where it is a commensal in the intestine (D’Alessandro et al., 2014; Davin-Regli and Pagés, 2015; Szczerba et al., 2020). *K. aerogenes* is also an opportunistic pathogen (Arpin et al., 1996; Davin-Regli and Pagés, 2015; Wesevich et al., 2020) that has caused several nosocomial outbreaks in the last few decades (Meyers et al., 1988; Allerberger et al., 1996; Arpin et al., 1996; Davin-Regli et al., 1996; Diene et al., 2013). This bacterium commonly produces respiratory infections, blood stream infections and urinary tract infections (UTIs) (Souza Lopes et al., 2016; Arpin et al., 2005; Arpin et al., 2003; Allerberger et al., 1996). Indeed, *K. aerogenes* is one of the most common causes of UTIs, besides uropathogenic *Escherichia coli* (Lodise et al., 2022). Complicated UTIs caused by *K. aerogenes* are often associated with resistance to commonly prescribed antibiotics, such as nitrofurantoin and third-generation cephalosporins (Lodise et al., 2022). *K. aerogenes* is able to adapt quickly during antibiotic treatment, causing therapy failure that is often fatal (Beckwith and Jahre, 1980; Petit et al., 1990; Chow, 1991; Diene et al., 2013). The number of cases caused by extended-spectrum beta-lactamase (ESBL)-producing *Enterobacterales*, including *K. aerogenes*, have alarmingly increased in the last 20 years, and the CDC has classified ESBL-producing *Enterobacterales* as serious threats that require prompt action (Centers for Disease Control and Prevention, 2019). Carbapenems have been widely used as antibiotic therapy against ESBL-producing pathogens, which quickly led to the emergence and propagation of carbapenem-resistant *Enterobacterales* (CRE), further reducing treatment options. In the US, *K. aerogenes* is one of the most common CRE (Karlsson et al., 2022) and the CDC has recently identified CRE microorganisms as public health threats that require urgent action (Centers for Disease Control and Prevention, 2019). In addition to beta-lactams and carbapenems, resistance to third-generation cephalosporins has been observed in a high percentage of isolated CRE *K. aerogenes* strains (Gashe et al., 2018), which produce nearly untreatable infections. The WHO (World Health Organization, 2017) has recognized *K. aerogenes* as a pathogen for which antimicrobial development is critically needed due to multidrug-resistance and its role in nosocomial infections (Mulani et al., 2019), ultimately leading to high risk of mortality and increased health care costs (Founou et al., 2017).

It is evident that new targets are urgently needed to treat infections caused by this pathogen. Targeting the molecular mechanisms used by the pathogen to survive and adapt to the host internal environment represents a novel alternative to produce antibiotics. However, little is known about the metabolism and physiology of *K. aerogenes*, or its metabolic adaptations in pathologically relevant conditions. Genomic analyses indicate that *K. aerogenes* codes for around 121 genes related to respiration or metabolism (Mann et al., 2021). However, the actual number and type of enzymes used in different conditions are almost completely unknown. Our group has previously described that respiratory enzymes in other bacteria, such as *Pseudomonas aeruginosa* (Liang et al., 2020) and *Chlamydia trachomatis* (Liang et al., 2018), are promising new targets for drug development, as these enzymes are not found in the human genome and their structural motifs are absent in human enzymes. Understanding the respiratory strategy of *K. aerogenes*, particularly in host-like conditions, may allow us to carry out drug design on specific targets that are essential for the growth and infectious process of this microorganism.

The aim of this work is to understand the respiratory pathway of *K. aerogenes* in conditions mimicking human urine. Our results show that this pathogen uses the type D_2_ NDH-2 dehydrogenase as the main NADH dehydrogenase and as the main entry point of electrons into the respiratory chain, and that the *bd*-I type terminal oxidase plays a major role in the electron transport chain. The data also show that this microorganism adapts its metabolism in urine-like conditions, increasing the expression of succinate dehydrogenase (SDH) and lactate dehydrogenase (LldD). These results provide essential information to understand the metabolic strategies used by this pathogen in colonization of the urinary tract and to propose alternatives for antibiotic treatment through the identification of targets for drug development.

## Materials and methods

### *In silico* screening of enzymes involved in respiration

Respiratory enzymes were identified in the *K. aerogenes* ATCC^®^ 35029 genome using a BLAST search approach. Sequences used as query were downloaded from Uniprot (Supplementary Table S1) and used in a local tBLASTn. Results were manually curated to eliminate false positives.

### Growth analysis

*Klebsiella aerogenes* ATCC 35029 growth behavior was assessed in LB and modified artificial urine media (mAUM), which was recently developed by our group (Liang et al., 2020). Growth curves were performed in 500 mL baffled flasks. Bacterial cultures were incubated at 37°C with shaking at 250 RPM. Samples were collected regularly, diluted and plated on LB plates; colony counts were determined after overnight incubation at 37°C. Experiments were performed at least three times on different days. To calculate the growth rate, maximum growth and lag phase length, growth curves were fitted to logistic functions as described previously (Liang et al., 2020).

### Isolation of *Klebsiella aerogenes* membranes

*Klebsiella aerogenes* ATCC 35029 cells were grown in either LB or mAUM. Bacterial cells were harvested by centrifugation (10,000 × *g* for 30 min at 4°C) during mid-log phase. Bacterial pellets were washed twice with KHE buffer (150 mM KCl, 20 mM HEPES, 1 mM EDTA, pH 7.5) and stored at −80°C. Frozen pellets were thawed on ice and homogenized in KHE buffer with 10 μg/mL DNAase I, 5 mM MgCl_2_ and 1 mM PMSF. Cells were lysed at 16,000 PSI using an Emulsiflex-C5 homogenizer. The cell lysate was centrifuged at 1,500 × *g* for 1 h to remove unbroken bacteria. Then the supernatant was spun at 48,000 × *g* for 1 h to remove cellular debris. Afterwards, the supernatant was centrifuged at 100,000 × *g* for 4 h to obtain the membrane pellet. The pellet was washed with KHE buffer and centrifuged at 100,000 × *g* for 1 h. The membrane pellet was resuspended in KHE buffer, aliquoted and stored at −80°C until further use.

### Oxygen consumption measurements

Oxygen consumption rates (OCR) of isolated *K. aerogenes* membranes were assessed using a YSI 5300 Clark-type electrode fitted to a 1.6 mL custom-made glass chamber. Oximetric analyses were performed using 0.1 mg/mL of protein in the presence of specific substrates and inhibitors for all respiratory complexes. NADH oxidase activity was measured using 200 μM NADH or 200 μM deamino-NADH with or without 1 μM rotenone. The activity of succinate dehydrogenase was measured using 20 mM succinate. For non-canonical ubiquinol-dependent respiratory enzymes, 10 mM L-lactate was used to measure the activity of lactate dehydrogenases, 10 mM D/L-malate for malate dehydrogenases, 10 mM D-glucose for glucose oxidases and 0.3% ethanol for alcohol dehydrogenases. Cytochrome *c* oxidase activity was measured using 100 μM TMPD (N,N,N′,N′-tetramethyl-p-phenylenediamine) and 5 mM ascorbic acid. Ubiquinol oxidase activity was evaluated using 50 μM of either ubiquinone-1 or menaquinone-2 reduced in the presence of DTT, as previously reported (Liang et al., 2020).

### KCN titration

The participation of terminal oxidases in the respiratory metabolism in *K. aerogenes* membranes was estimated carrying out a titration with KCN (0.1–10 mM). The titration curve was analyzed as previously described by our group (Liang et al., 2020), fitting the data to a two-component equation, assuming that the concentration of substrates is near saturation ([S]/Km > 10).

### Blue native gel electrophoresis

Membrane samples were washed and resuspended in Buffer M (50 mM Bis-Tris, 500 mM aminocaproic acid, pH 7.0). Membranes were solubilized using Triton X-100 at a protein: detergent ratio of 1:2. The samples were incubated on ice for 40 min and centrifuged at 120,000 × *g* for 40 min. Solubilized membranes were mixed with Cathode Buffer I (50 mM Tricine, 15 mM Bis-Tris, 0.02% Coomassie Brilliant Blue G-250, pH 7.0) at a 5:1 ratio and applied to a 4–16% gradient native polyacrylamide gel, as described previously (Liang et al., 2020). For NADH activity, gels were incubated in NADH Reaction Buffer (100 mM Tris–HCl, 140 μM NBT, pH 7.4) for 1 h.

### Proteomic analysis

LB or mAUM membranes were solubilized in 1% SDS and centrifuged at 13,000 × *g* to remove non-solubilized debris. Protein preparation and analysis was performed by The Mass Spectrometry Core of the Research Resources Center of University of Illinois at Chicago. 50 μg of protein per sample were filtered using 10 K NMWL centrifugal filter units. Proteins were reduced by incubation in 20 mM dithiothreitol for 30 min and were alkylated with 50 mM iodoacetamide for 20 min in the absence of light. Samples were washed three times using buffer containing 8 M urea and 0.1 M ammonium bicarbonate. Afterwards, washed samples were equilibrated with 0.1 M ammonium bicarbonate and transferred to a buffer containing 0.1 M ammonium bicarbonate and trypsin at an enzyme: protein ratio of 1:20. The digestion was incubated at 37°C overnight. Peptides were recovered by centrifugation at 14,000 × *g* for 20 min and eluted twice with 50 mM ammonium bicarbonate with an additional elution using 0.5 M NaCl. Finally, samples were desalted with an Oasis Prime HLB and then dried. Digested samples were labeled using TMT 6plex isobaric Label Reagent Set (ThermoFisher, Waltham, MA, USA) following manufacturer’s instructions. Samples were combined, dried and desalted using a primed HLB 96 well plate. Off-line high-HP reverse phase liquid chromatography was performed to obtain fractions which were concatenated in 6 pools. Pooled fractions were dried and resuspended in 5% acetonitrile and 0.1% formic acid buffer for LC–MS analysis. Mass spectrometry data were searched against the Uniprot database of *K. aerogenes* proteins using Mascot Engine (2.6.0) with a parent mass tolerance of 10 ppm, a constant modification on cysteine alkylation, variable modification on methionine oxidation, deamination of asparagine and flutamine, and TMT purity correction. Results were analyzed in Scaffold Q + S Software v 5.0.0 (Proteome software, Portland, OR, USA) using filtering criteria of 1 minimum peptide count and a false discovery rate of 1%.

## Results

### *Klebsiella aerogenes* growth in urine-like medium

**Figure 1:**
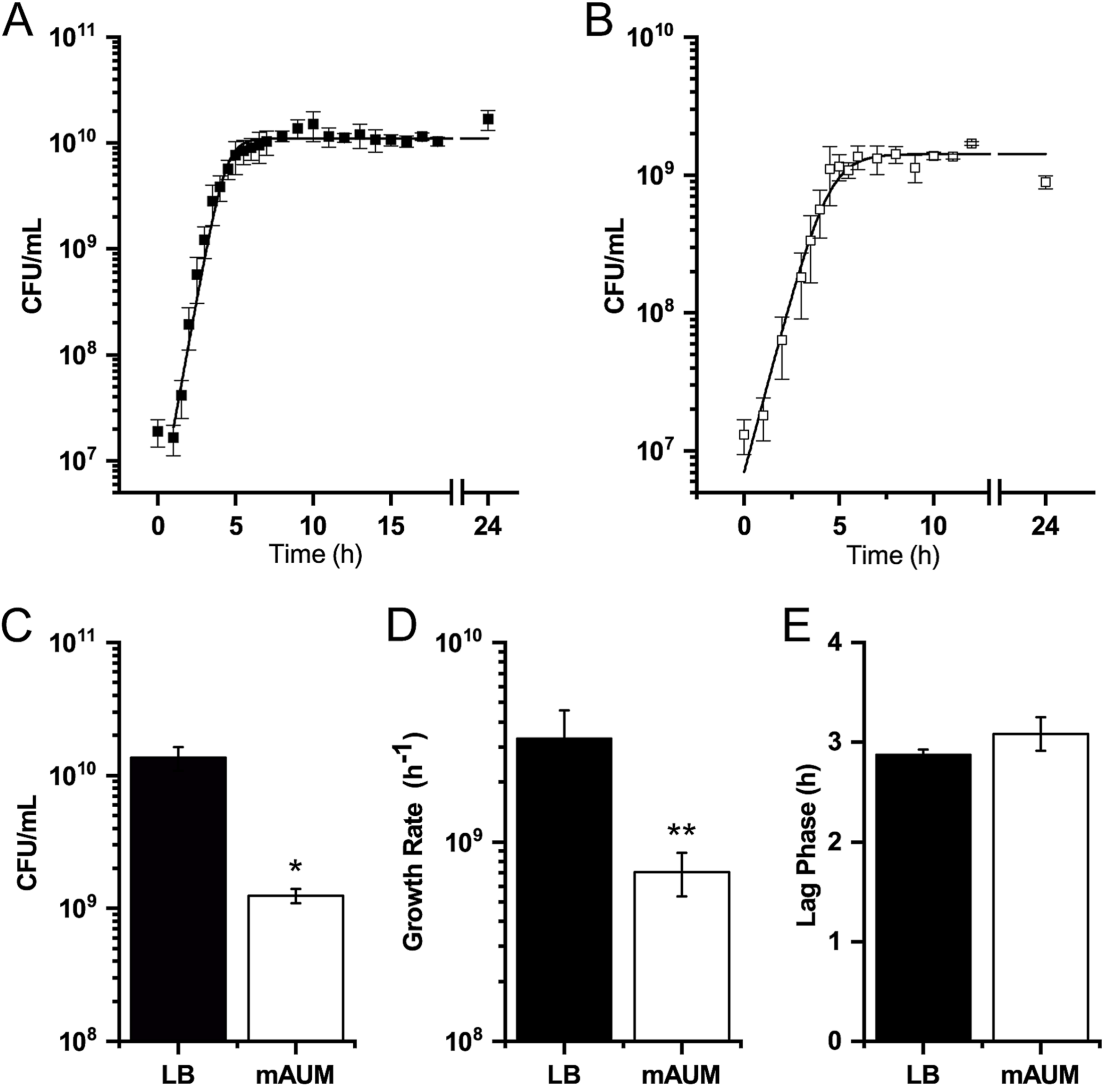
*K. aerogenes* growth in LB and mAUM. **(A)** Growth curve in LB broth. **(B)** Growth curve in mAUM. **(C)** Maximum growth. **(D)** Growth rate. **(E)** Duration of lag phase. Black bars represent data from LB and white bars indicate data from mAUM. Data are expressed as the mean ± SD, *n* = 3. The asterisk indicates significance mAUM vs. LB (*p < 0.05, **p = 0.001) determined by *t*-test analysis.

In this work, we characterized the growth of *K. aerogenes* in media that mimics human urine. For this purpose, we employed the previously described mAUM, developed by our group, which offers more stability and reliability compared to other urine-like media (Liang et al., 2020). There is no information regarding the growth behavior of *K. aerogenes* in standard laboratory media or physiologically relevant conditions, for this reason the results obtained in urine-like media were compared to the enriched laboratory media, LB broth (Figure 1). This helped us to understand the general metabolic pathways used by this pathogen and the adaptations to urine-like media. This organism was able to grow in both enriched and urine-like media, which in the constant agitation conditions used remained 80–85% oxygen saturated (measured with a Clark-type oxygen electrode, as described below). *K. aerogenes* showed typical sigmoidal growth in both media analyzed (Figures 1A,B). We observed that media composition influenced growth behavior, with higher bacterial counts in LB media (CFU/mL) than in mAUM (Figure 1C). In contrast to growth in enriched media, *K. aerogenes* displayed a slower growth rate and lower bacterial density in mAUM (Figure 1D), but no difference in the lag phase (Figure 1E), suggesting that this bacterium is able to quickly adapt to growth using components present in human urine. These results indicate that *K. aerogenes* carries the metabolic machinery that allows its adaptation during growth in urine and likely during urinary tract infections.

### *Klebsiella aerogenes* respiratory genes

**Figure 2:**
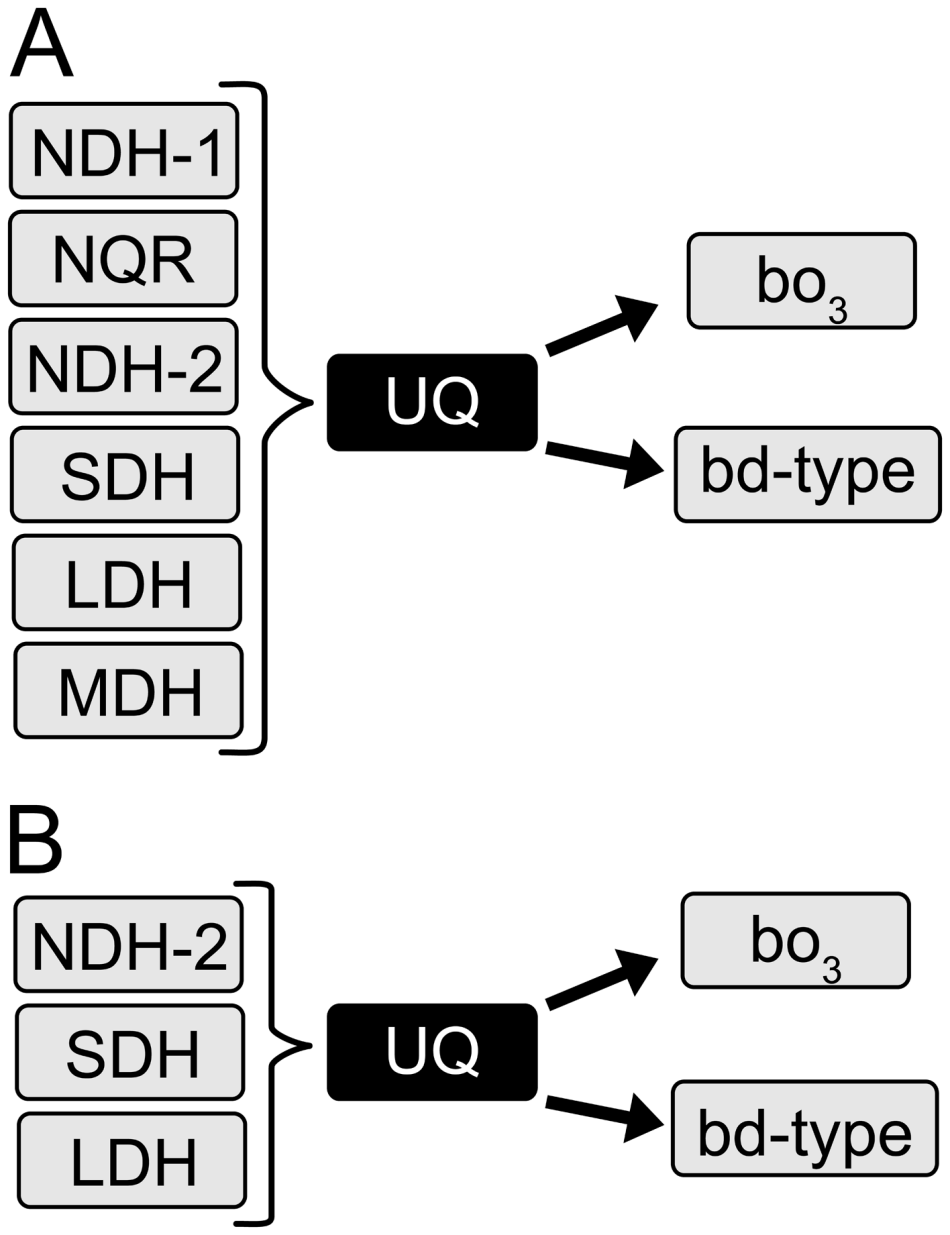
Respiratory chain of *K. aerogenes*. **(A)** Respiratory enzymes present in the *K. aerogenes* genome. **(B)** Main respiratory enzymes used by *K. aerogenes* during growth in LB and mAUM.

We identified and classified the genes involved in respiratory metabolism in the *K. aerogenes* genome from the NCBI repository, using different query sequences of each known membrane protein involved in aerobic respiration. Our search shows that the *K. aerogenes* genome contains genes that code for three NADH: ubiquinone oxidoreductases: NDH-1 (complex I-type), NDH-2 (two class D_2_ and one class C enzymes) and NQR (Figure 2). NDH-1 is a proton-pumping dehydrogenase, composed of 13–14 subunits with one FMN and multiple Fe-S centers (Brandt, 2006). NQR is a six subunit, six cofactor (Juárez et al., 2008; Juárez and Barquera, 2012; Reyes-Prieto et al., 2014) respiratory enzyme that pumps sodium (Bogachev et al., 1997; Barquera et al., 2002), and at least in one case protons (Raba et al., 2018). NDH-2 is a single subunit and single cofactor dehydrogenase that does not pump ions and does not contribute to cell energetics (Ito et al., 2020). Other dehydrogenases found in the genome are succinate dehydrogenase (SDH), three membrane-bound NAD-independent lactate: quinone oxidoreductases (LldD, DLD and LdhA) and two membrane-bound malate: quinone oxidoreductases (MQO1 and MQO2) (Figure 2). Moreover, we determined that this bacterium carries the genes coding for one cytochrome *bo*_3_ terminal oxidase and four *bd*-type terminal oxidases. For the *bd*-type oxidases, two operons belong to the *bd*-I type and two to the *bd*-II type terminal oxidases. Furthermore, we found the genes involved in the biosynthesis of ubiquinone and the partial biosynthetic pathway of menaquinone, with the absence of MenA and MenG, which indicate that this quinone is not biosynthesized by *K. aerogenes*. This is in accordance with previous reports, which show that ubiquinone-8 is the sole quinone present in *K. aerogenes* under aerobic and anaerobic conditions (Knook and Planta, 1971, 1973). Finally, *K. aerogenes* encodes the machinery for biosynthesis of cytochrome *c*. However, it does not seem to have a role in aerobic respiration, as the *bc*_1_ complex and cytochrome *c* oxidases are not found in the genome.

### *Klebsiella aerogenes* metabolic repertoire is more active during growth in urine-like conditions

**Figure 3:**
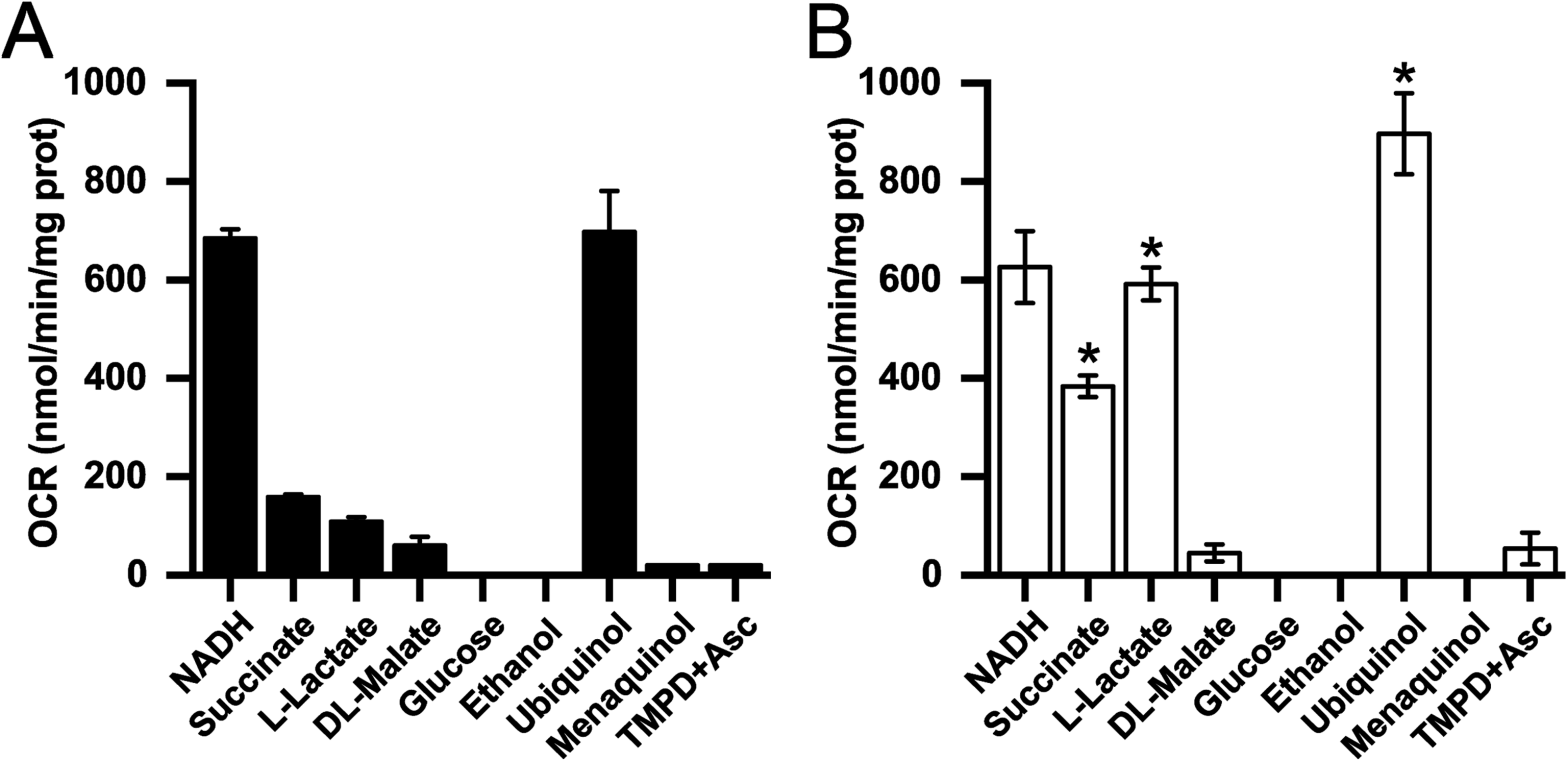
Oxygen consumption rate (OCR) of isolated *K. aerogenes* membranes in the presence of different substrates. Membranes isolated from cells grown in LB **(A)** or mAUM **(B)**. Data are expressed as average ± S.D. Black bars show LB data and white bars show mAUM data. *n* ≥ 3. Asterisks indicate significance between LB and mAUM (*p < 0.05) determined by *t*-test analyses.

To identify the metabolic adaptations that *K. aerogenes* uses to grow in mAUM, we analyzed oxygen consumption rates in membranes using substrates of different enzymes involved in respiration. Our results show that oxygen consumption activity is higher in membranes obtained from cells grown in mAUM compared to LB (Figure 3), suggesting that the respiratory chain plays a major role in the adaptation to urine and likely in UTIs. In mAUM membranes, succinate dehydrogenase and lactate dehydrogenase activities showed a 2.5-and 4.4-fold increase, respectively, suggesting that *K. aerogenes* adapts its metabolism and uses the substrates present in urine to grow efficiently during UTIs. Although there were significant differences in the growth parameters, we found no difference in the NADH dehydrogenase or malate dehydrogenase activities in both conditions. Glucose oxidase and ethanol dehydrogenase activities were negligible, indicating that these substrates are not used by the electron transport chain.

In addition to the dehydrogenases, we also studied the quinone preference of *K. aerogenes* respiratory machinery using ubiquinol-1 and menaquinol-2 as substrates for the terminal oxidases. Our results show that the terminal oxidases of cells grown aerobically, preferentially use ubiquinol in both mAUM and LB media. While LB membranes were able to use menaquinol at low rates, no menaquinol oxidase activity was detected in mAUM (Figure 3). Additionally, we found that the activity with TMPD and ascorbate is negligible, indicating that this microorganism does not use cytochrome *c* oxidases, confirming the results obtained from the genome analysis.

### NDH-2 as the main NADH dehydrogenase in *Klebsiella aerogenes*

**Figure 4:**
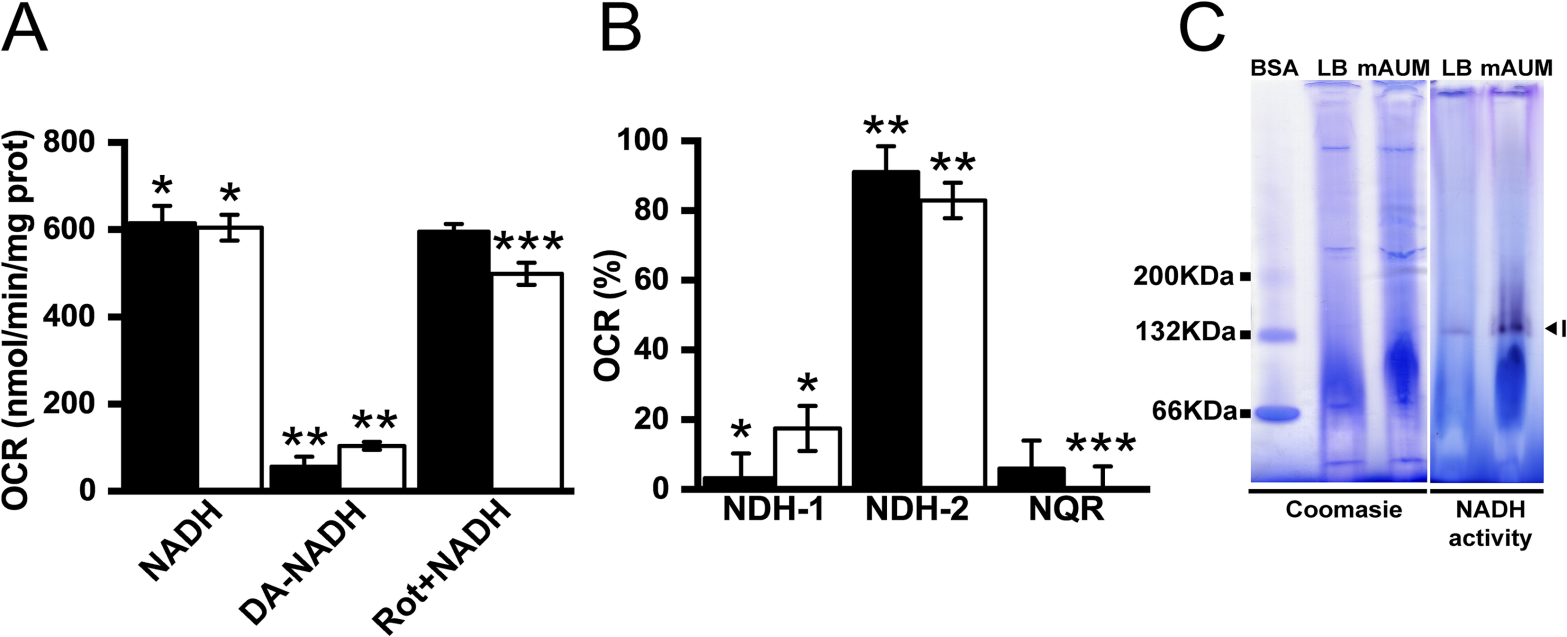
NADH dehydrogenases in *K. aerogenes* membranes. **(A)** Oxygen consumption rate using NADH, deamino-NADH (DA-NADH) and rotenone (Rot). **(B)** Participation of NADH dehydrogenases in oxygen consumption rates. **(C)** NADH dehydrogenase activity observed in blue native gel. Label I indicates a band of approximately 132 KDa (arrow head). BSA in its different oligomeric states is used as weight marker. Black bars correspond to data obtained from LB and white bars from mAUM. Data are expressed as average ± SD. *t*-test statitical analyses were performed for panels **(A,B)** (*p* < 0.05), asterisks correspond to comparison between groups: **(A)** *NADH vs. DA-NADH; **DA-NADH vs. Rotetone; ***NADH vs. Rotenone. **(B)** *NDH-1 vs. NDH-2; **NDH-2 vs. NQR; ***NDH-1 vs. NQR.

Although NDH-1, NDH-2 and NQR catalyze the same chemical reaction, they can be differentiated by substrate specificity and inhibitor selectivity. NDH-1-type dehydrogenases employ NADH and deamino-NADH as substrates, and are susceptible to inhibition by rotenone (Yagi, 1991; Zickermann et al., 2000; Melo et al., 2004). NQR can also use NADH and deamino-NADH as substrates, but it is insensitive to rotenone (Zhou et al., 1999; Hreha et al., 2021). NDH-2 is insensitive to rotenone and can only use NADH as substrate (Yagi, 1991; Melo et al., 2004; Hreha et al., 2021). As shown in Figure 4A, the deamino-NADH oxidase activity in membranes is <10% in LB and < 20% in mAUM compared to the NADH oxidase activity, indicating that NDH-2 is the main NADH dehydrogenase in this organism (Figure 4B). Rotenone does not significantly inhibit the deamino-NADH independent activity and thus we can assign NQR as the second most important dehydrogenase in LB. In mAUM, the NDH-1 activity is higher compared to the activity of NQR. To corroborate the activity data, we conducted Blue Native Gel electrophoresis analysis of the respiratory complexes in membranes solubilized with 1% Triton X-100. The three NADH dehydrogenases can also be differentiated by their electrophoretic mobility in native gels, as NDH-1, NQR and NDH-2 have estimated molecular weights of 550 kDa (Yip et al., 2011), 200 kDa (Juárez and Barquera, 2012) and 47 kDa (Yagi, 1991), respectively. As shown in Figure 4C, the samples ran in BN-PAGE contain a single band with NADH dehydrogenase activity of approximately 132 kDa. This band is too small to correspond to NDH-1 or NQR and likely corresponds to an NDH-2 dimer. The presence of a dimeric NDH-2 has been previously reported in other organisms, including *Caldalkalibacillus thermarum* (Feng et al., 2012; Heikal et al., 2014; Nakatani et al., 2017).

**Figure 5:**
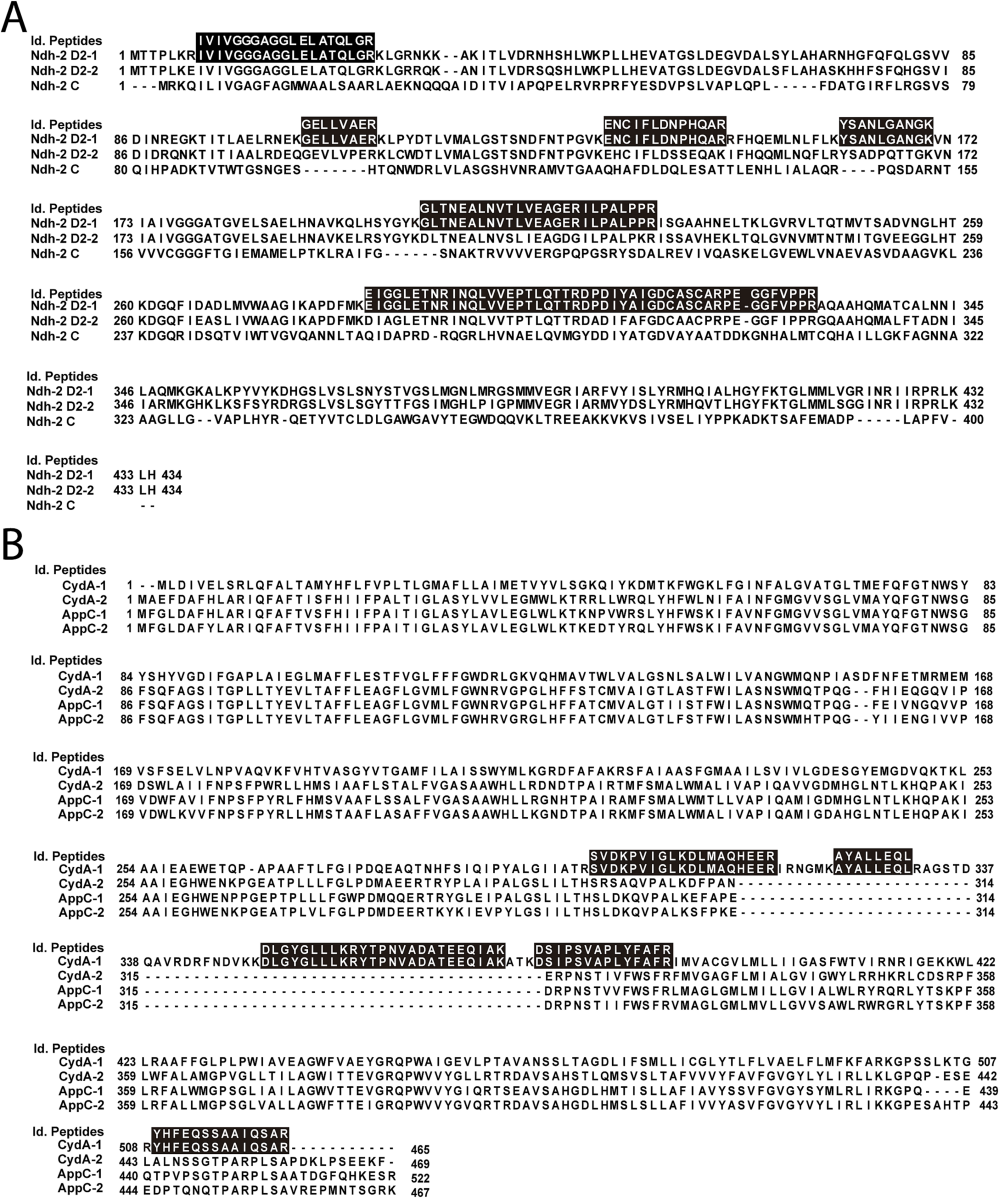
Sequence analysis of peptides. **(A)** Alignment of the *K. aerogenes* NDH-2 dehydrogenases and the peptides identified by proteomic analysis. **(B)** Alignment of subunit I of *K. aerogenes bd*-type terminal oxidases. Black boxes indicate the sequence of the peptides found in proteomic analysis. Alignment of amino acid sequences was performed on ClustalX2.

As described above, *K. aerogenes* carries three NDH-2 genes, two class D_2_ and one class C. To identify the main NDH-2 enzyme involved in electron transfer, we carried out proteomic analysis of membranes obtained in LB and mAUM. As shown in Figure 5A, mass spectrometry identified peptides belonging exclusively for NDH-2 D_2_-1, indicating that this is the NDH-2 responsible for the NADH dehydrogenase activity and most of the NADH-dependent respiration in this microorganism.

### *Bd*-type oxidases are the main terminal oxidases in *Klebsiella aerogenes*

**Figure 6:**
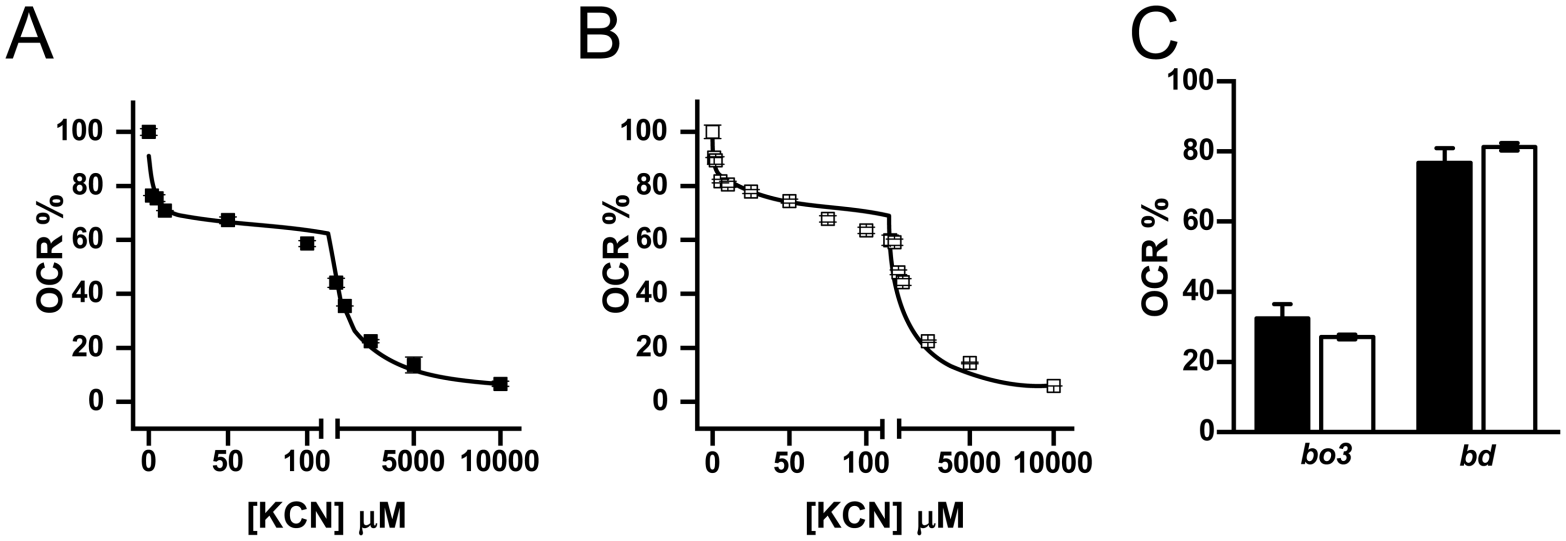
Contribution of terminal oxidases to the respiratory activity of isolated *K. aerogenes* membranes. Oxygen consumption rate was measured under increasing concentrations of KCN. KCN tiration of LB **(A)** and mAUM **(B)** membranes using a two-component formula (see material and methods). **(C)** Relative contribution of *bo*_3_ and *bd*-type oxidases to the respiratory activity of LB (black bars) or mAUM (white bars). Data expressed as average ± S.D. *n* ≥ 3.

To elucidate the contribution of terminal oxidases to the respiratory metabolism in *K. aerogenes*, a KCN titration of the respiratory activity was carried out. Testing the cyanide susceptibility of the respiratory activity is a useful method to estimate the participation of the terminal oxidases, as the reported *Ki_app_* for KCN of the terminal oxidases differs by more than one order of magnitude. For instance, the *Ki_app_* of *bo*_3_ oxidases is significantly lower than *bd* oxidases, typically ranging from 10 to 40 μM (Mogi et al., 2009; Weiss et al., 2010; Melin et al., 2013; Liang et al., 2020), although significant variability has been observed (see below), while the *Ki_app_* of *bd*-oxidases ranges from 1 to 30 mM (Matsushita et al., 1983; Forte et al., 2017). The titration data obtained for both types of membranes was best fitted to a two-component inhibition model (Figure 6). The first component, representing 27–32% of the activity in both types of membranes, has a *Ki_app_* of 0.2 ± 0.1 μM in LB and 0.3 ± 0.05 μM in mAUM membranes, likely corresponding to *bo*_3_-type oxidases. The second component has a *Ki_app_* of 106 ± 14 μM in LB and 123 ± 2 μM in mAUM, accounting for 76–81% of the respiratory activity and likely corresponding to *bd*-type oxidases. Thus, our results indicate that *bd*-oxidases are the main terminal oxidases in *K. aerogenes*, and for this reason, we decided to carry out proteomic analysis to identify the main enzyme.

As described above, *K. aerogenes* carries two *bd*-I (*cydABX*-1 and *cydABX*-2) and two *bd*-II oxidase operons (*appCBX*-1 and *appCBX*-2), as well as one *bo*_3_ operon (*cyoABCDE*). In order to identify the specific enzymes expressed by this pathogen in mAUM and LB media, we performed proteomic analysis of isolated membranes from bacteria grown in the two different media (Figure 5). Proteomic analysis demonstrates the presence of subunits CyoA and CyoB of the *bo*_3_ oxidase, as the next peptides were identified for CyoA (accession number A0A0F1LA05): GQIGLEQR, YSPNWSHSNK, VTSNSVMNSFFIPR, ATFDQWVAK, QSPNSMDSMAAFDK and VAVPSENNK, and for CyoB (accession number A0A44LCJ9): APGMTMFK, AFGFTLNETWGK, DLTGDPWGGR, TLEWATSSPPPFYNFAVVPNVHER, QPAHYEEIHMPK, SFDEDVDYYVPVAEVEK and LENQHFDEINK. Our data also show that of the four *bd*-type enzymes, only the *bd*-I encoded in the *cydABX*-1 operon was expressed in these membranes (Figure 5B). This operon is located in a genetic locus similar to the *E. coli cydABX* operon, which is differentiated from other *cydABX* operons by the presence of the *ybgE* and *ybgC genes*, flanked by *sdhCDAB*, *sucABCD* and *tolQRAB* operons (Muller and Webster, 1997). In *E. coli*, *cydABX* codes for the main terminal oxidase in this microorganism during the stationary phase and under microaerophilic conditions (Tseng et al., 1996; Grund et al., 2021).

### Proteomic analysis of *Klebsiella aerogenes* membrane associated transporters and respiratory *enzymes*

**Figure 7:**
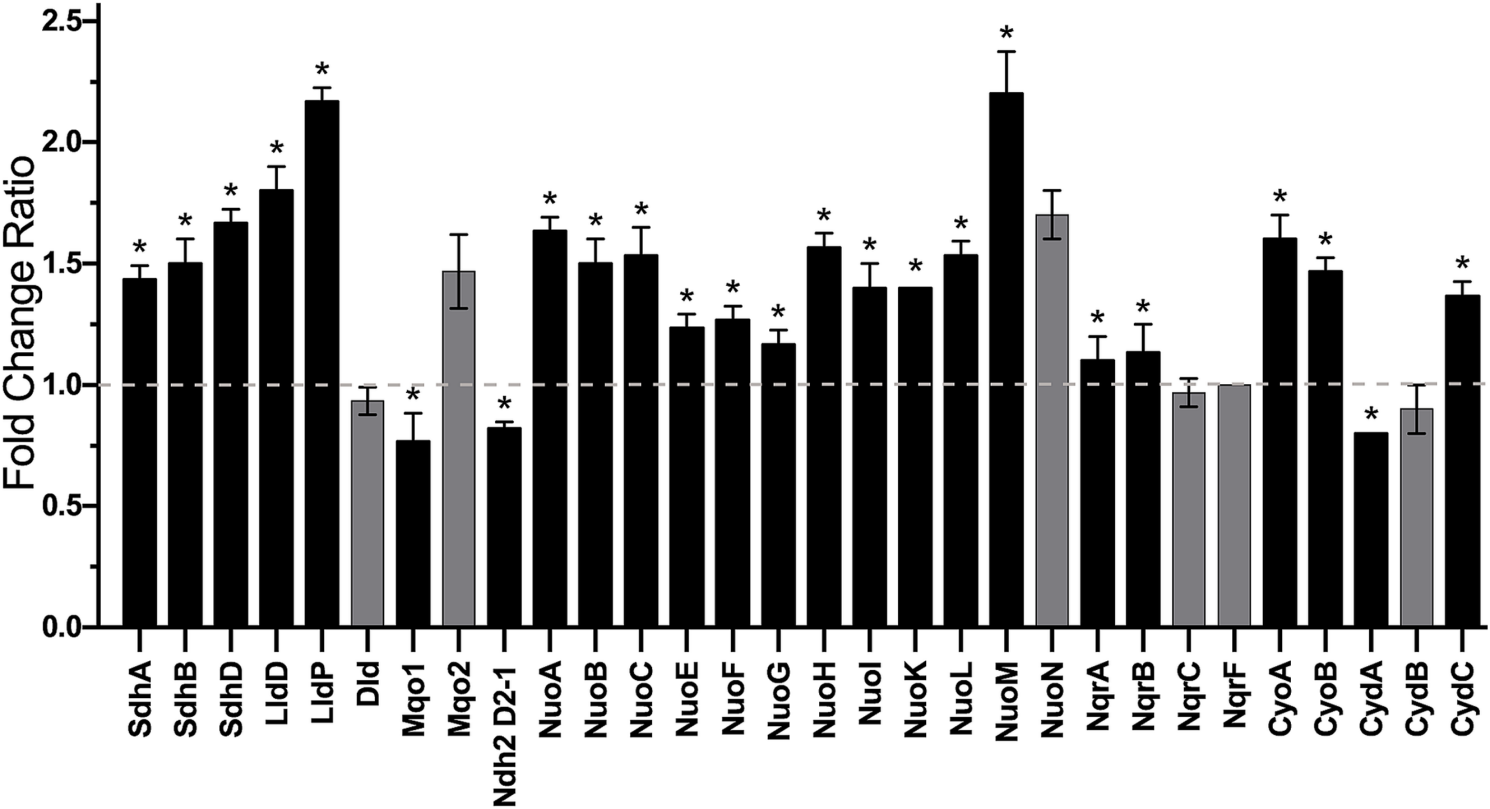
Proteomic data of main NADH-dehydrogenases, NADH-independent ubiquinone-dependent dehydrogenases and terminal oxidases. Fold change ratio of mAUM data vs. LB data is shown. Dashed line indicates no change. Data represent average ± S.D., *n* = 3. Asterisks denote significance (*p* = 0.01) as determined by *t*-test. Non-significant change is represented by gray bars.

A proteomic analysis was performed to propose the metabolic machinery employed by *K. aerogenes* during growth in urine-like conditions by comparing the content of respiratory enzymes and membrane associated transporters of membranes of cells grown in LB and mAUM. As indicated above, NDH-2 is the main NADH-dehydrogenase in both conditions (Figure 4). Although oximetry data did not show a significant change in the overall NADH dehydrogenase activity in mid-log growth among both conditions, the content of the different enzymes changed in mAUM when compared to LB as determined by proteomic analysis: the NDH-2 (D2-1 isoform) showed a small decrease (0.8-fold change ratio), a significant increase (1.5 fold-change on average) of NDH-1 was observed as determined for Nuo proteins, and we also observed a small increase (11.5% average) in NQR subunits (Figure 7). *K. aerogenes* also seem to adapt its metabolism to utilize components present in host-like conditions (mAUM). SDH and the membrane-bound NADH-independent L-lactate dehydrogenase (LldD) showed a significant increase in expression and activity in mAUM (Figures 3, 7, respectively). SDH-dehydrogenase activity increased dramatically during growth in urine-like conditions and this correlates with an average fold-increase of SDH proteins of 1.5 (Figure 7). Citrate and *α*-ketoglutarate are molecules that enter the Krebs cycle which in turn increase the amount of succinate available for the SDH. Interestingly, a system involved in citrate uptake (Kästner et al., 2002; Scheu et al., 2012) was also upregulated, we found the sensor histidine kinase CitA (1.5 fold change ratio), and the citrate-acetate antiporter CitW (3.4-fold change) content increased in mAUM compared to LB (Figure 7). Moreover, the α-ketoglutarate transporter KtgP was also upregulated in mAUM (1.2-fold increase) (Figure 7). We also found an increase in the expression of LldD (1.8 fold) and the lactate transporter LldP (2.1-fold), indicating higher use of lactate in mAUM compared to LB. However, we found no changes in another membrane-bound LDH (Dld). Furthermore, of the two membrane-bound malate dehydrogenases (MQO) coded in the genome, we observed a change of 0.6 for Mqo1 and no changes in the content of Mqo2 (Figure 7). However, differences in the activity of malate-dependent quinone-oxidoreductase were not observed during oximetry analysis (Figure 3). Finally, changes in the expression of terminal oxidases were analyzed. The activity of *bo*_3_ and *bd*-I terminal oxidases was similar in both media employed. However, we found an average 1.5 fold-increase in expression of *bo*_3_ in urine-like conditions (Figure 7). On the other hand, the expression of CydA, structural part of the *bd*-I complex, showed a small decrease (0.8 fold-change) while CydC, which is involved in the biogenesis of the *bd*-I enzyme, exhibited a 1.2 fold-increase (Figure 7). Altogether, proteomic and oximetric data suggests that *K. aerogenes* undergoes metabolic changes to adapt to growth in host-like fluids, utilizing the available substrates present.

## Discussion

Opportunistic emerging pathogens are highly relevant in clinical settings due to their capacity to survive antibiotic treatment, which greatly complicates patient outcomes (Beckwith and Jahre, 1980; Petit et al., 1990; Chow, 1991; Diene et al., 2013; Wesevich et al., 2020). Among these pathogens, *K. aerogenes* has become increasingly problematic as it is resistant to first line antibiotics, and in some cases even to last resort drugs in pandrug-resistant strains (Tuon et al., 2015; Wesevich et al., 2020; Pan et al., 2021). Due to the number of antibiotic resistant infections, the CDC has ranked CRE, including several strains of *K. aerogenes*, as a priority for the development of new antibiotics (Centers for Disease Control and Prevention, 2019). Studying the metabolic adaptations of pathogenic bacteria is essential for the development of novel strategies to combat antibiotic resistance, as it highlights relevant enzymes that could be employed as drug targets. However, most studies are performed in conditions that are not relevant for the pathophysiology of the microorganism and do not simulate the host environment, potentially misguiding the selection of targets. In fact, there are no studies that describe *K. aerogenes* metabolism in conditions that are relevant in the clinical setting. In this work, we carried out a systematic characterization of the *K. aerogenes* respiratory chain in standard laboratory conditions and in conditions that simulate a common site of infection, the urinary tract. Studying the growth and metabolism of *K. aerogenes* in mAUM, we obtained data from conditions that closely resemble the environment in the urine, which has led us to identify critical enzymes that could be used to produce the desperately needed novel antibiotics.

### Growth dynamics in urinary media

Most research involving *K. aerogenes* is focused on fermentation using different carbon sources (Kucharska et al., 2019; Wu et al., 2021; Boonyawanich et al., 2023), with little to no information regarding clinically relevant conditions. In nature and during infection, bacteria are rarely in an environment with optimal conditions for growth, therefore results obtained in laboratory media, like LB broth, do not represent the behavior within the host. Thus, we decided to investigate the respiratory metabolism of *K. aerogenes* in media that mimics urine, in which the bacterium should have significant adaptations, as previous studies have shown that bacterial pathogens carry specific sets of genes for the adaptation of bacteria to these environments (Frick-Cheng et al., 2020; Liang et al., 2020).

*Klebsiella aerogenes* showed a typical growth curve in both LB media and in mAUM. While *K. aerogenes* biomass and growth rate are lower compared to LB media, this organism can still grow significantly in mAUM. Urine-like conditions impose a metabolic and physical burden due to high salinity, low pH, and low nutrient content and composition. However, *K. aerogenes* can still grow in these conditions, suggesting that this pathogen can replicate efficiently during urinary tract infections. Our results also show that *K. aerogenes* may reach stationary phase relatively fast during growth in urine-like conditions. This becomes relevant as tolerance to antibiotics develops during this phase, when bacterial growth rate and metabolism are markedly reduced (Brauner et al., 2016), which could be another factor that contributes to the intrinsic resistance of *K. aerogenes* to antibiotics.

### *Klebsiella aerogenes* respiratory adaptations in urine-like conditions

mAUM, a medium recently developed by our group that closely resembles the composition of human urine, is more stable and reliable compared to other AUM (Brooks and Keevil, 1997; Liang et al., 2020). Moreover, we have previously shown that it supports the growth of *P. aeruginosa,* a common pathogen of the urinary tract and catheter-associated urinary tract infections (Rossolini and Mantengoli, 2005; Mittal et al., 2009; Liang et al., 2020; Ronald, 2002). mAUM is a relatively nutrient-poor medium that, as shown here, can also support the growth of *K. aerogenes*, which switches its metabolism in order to survive in this environment by significantly activating succinate and lactate dehydrogenases. Urine-like mAUM contains physiological amounts of L-lactate (Kramer et al., 1972) and citrate (Wein et al., 2011), thus the activation of membrane-bound lactate dehydrogenases and the Krebs cycle is expected. Interestingly, other pathogenic bacteria do not use the same metabolic strategies found here (Liang et al., 2020). For instance, our group has previously studied *P. aeruginosa* under similar conditions and we have found that SDH and LDH activities were not increased in mAUM (Liang et al., 2020), highlighting the importance of studying each pathogen independently.

### *Bd* oxidase as the main terminal oxidase in *Klebsiella aerogenes*

Bioinformatics analysis showed that *K. aerogenes* carries one *cyoABCDE* operon and four *bd*-type terminal oxidase operons, two for type I *bd* and two for type II *bd* enzymes, making this the highest number of *bd*-type oxidases reported, with most microorganisms carrying only one operon (Forte et al., 2017). Our results show that *bd*-oxidases carry out 76–81% of the respiratory activity in *K. aerogenes*, a different metabolic strategy compared to other microorganisms. In other bacteria, *bo*_3_ enzymes are the terminal oxidases preferentially used in oxygen-rich environments (Tseng et al., 1996). Indeed, we have reported that in *P. aeruginosa*, which carries the three types of terminal oxidases described above, *bo*_3_ oxidase acts as the main terminal oxidase (Liang et al., 2020), resembling the case of *E. coli* and *Gluconobacter*, where *bo*_3_ oxidase has also been reported as the main oxidase (Tseng et al., 1996; Richhardt et al., 2013). Thus, our results show that the metabolic strategies used by *K. aerogenes* are different compared to those of other pathogens.

Our proteomic results show that the *bd*-I oxidase is used by *K. aerogenes* during logarithmic growth. However, *bd*-II enzymes may be employed by *K. aerogenes* in other environmental conditions, providing a wide repertoire that allows adaptation to different environments. While the role of terminal oxidases in pathogenesis remains unclear, it has been proposed that *bd*-I oxidases are expressed in conditions where oxygen tension is below 50% (Grund et al., 2021). Additionally, it has been reported that *bd*-type enzymes act as ROS scavengers. Indeed, in *E. coli*, *bd*-type enzymes have peroxidase-like and catalase-like activities (Borisov et al., 2011a; Lu et al., 2015; Al-Attar et al., 2016; Forte et al., 2017; Borisov et al., 2021), which could be a mechanism of defense against the immune system and a factor that might determine pathogenicity. Our data indicate that *K. aerogenes* uses cytochrome *bd*-I oxidase encoded by the operon *cydABX*-1 as the main terminal oxidase. *Bd*-I oxidases could be important for *K. aerogenes* as they might contribute to the rapid growth and colonization of human tissues during infection. In *Salmonella*, *bd*-I oxidases appear to provide a fitness advantage during the colonization of mouse tissues (Rivera-Chávez et al., 2016). On the other hand, in *E. coli*, *bd*-II oxidases are expressed upon entry to stationary phase and by phosphate starvation (Brøndsted and Atlung, 1996; Grund et al., 2021), suggesting that these enzymes could be more relevant during the switch to anaerobic growth (Brøndsted and Atlung, 1996; Tseng et al., 1996; Grund et al., 2021). *Bd*-II type oxidases have also been linked to fitness advantages following antibiotic treatment in mice (Rivera-Chávez et al., 2016).

Our results also show that *K. aerogenes* terminal oxidases have different sensitivities to cyanide compared to other enzymes of the same family. In most instances, *bd* oxidases tolerate high cyanide concentrations. For example, in *P. aeruginosa* the cyanide concentration needed to reach 50% inhibition (IC_50_) ranges from 3 to 30 mM, depending on growth conditions (Matsushita et al., 1983; Forte et al., 2017). For *E. coli*, a concentration of 2 mM is required to observe the same effect (Forte et al., 2017; Liang et al., 2020). However, these values are highly variable and there are instances where *bd* oxidases are not severely tolerant to cyanide. For instance, the *bd* oxidases of *Photobacterium phosphoreum* and *Geobacillus thermodenitrificans* have IC_50_ of 62 μM and 500 μM, respectively (Konishi et al., 1986; Sakamoto et al., 1999), within the range that we are reporting for *K. aerogenes*, 150 μM. These results suggest that *K. aerogenes bd*-type enzymes carry differences in structure that make them more susceptible to inhibition by KCN compared to other enzymes. Interestingly, *bd*-type enzymes are only present in prokaryotic organisms and are associated with colonization of host tissue (Rivera-Chávez et al., 2016), making them an attractive target for drug development (Forte et al., 2017). Their differences in KCN sensitivity may suggest that they could be differentially inhibited by newly designed drugs without affecting *bd*-type enzymes from the host microbiota, offering an important advantage compared to traditional antibiotics.

### NDH-2 is the main NADH dehydrogenase in *Klebsiella aerogenes*

In this work, we are describing that *K. aerogenes* NDH-2 likely forms homodimers, as has been reported in other cases (Heikal et al., 2014). Interestingly, in other organisms NDH-2 can also from supercomplexes with other respiratory enzymes, such as lactate dehydrogenases and ATPase subunits (Grandier-Vazeille et al., 2001). Moreover, proteomic analysis demonstrates that *K. aerogenes* employs a D_2_ class NDH-2, which is different compared to *M. tuberculosis* D4 and *S. aureus* D1 classes (Marreiros et al., 2016). Our group and others have demonstrated that NQR is the main NADH dehydrogenase in many pathogenic microorganisms such as *P. aeruginosa* (Liang et al., 2020), *Vibrio cholerae* (Tuz et al., 2015; Agarwal et al., 2020), *C. trachomatis* (Liang et al., 2018), *Bacteroides fragilis* (Ito et al., 2020) and *Prevotella* spp. (Deusch et al., 2019), maintaining the motive force across the membrane and providing energy for ATP production and other vital processes. However, *K. aerogenes* does not seem to fit within this seemingly emerging trend in pathogens. We found that in *K. aerogenes*, NDH-2 is the main entry of electrons into the respiratory chain in the conditions tested, which represent widely different environments, while NDH-1 and NQR contribute to a small fraction of NADH-dehydrogenase activity. Previous works have shown that deletion of the NDH-1 genes *nuoD* and *nuoC* do not impact *K. aerogenes* growth (Wu et al., 2021), corroborating the data found here. In addition to *K. aerogenes*, other pathogens also rely heavily on NDH-2 in their respiratory metabolism, especially in those where NQR is absent (Schurig-Briccio et al., 2014; Lencina et al., 2018; Beites et al., 2019). For instance, in *Streptococcus agalactiae* the elimination of NDH-2 heavily impacts its capacity to colonize mouse kidney (Lencina et al., 2018). NDH-2 is a key enzyme during growth in enriched or in nutrient-limited conditions, suggesting that its loss or inhibition may critically impair *K. aerogenes* growth, causing cell death or allowing other drugs to exert their effects more efficiently, which opens opportunities to identify and design new drugs that target this enzyme against extensively drug-resistant or pan-resistant *K. aerogenes* strains.

### *Klebsiella aerogenes* metabolism in urine-like media

*Klebsiella aerogenes* is a common constituent of the normal human microbiota and in certain conditions, it can become a facultative pathogen, producing one of the most common types of multidrug resistant UTIs (Lodise et al., 2022). In this work, we have elucidated for the first time the metabolic adaptations of this pathogen for the growth in urine-like media and likely during UTIs. *K. aerogenes* is able to employ the components present in mAUM, particularly citrate and lactate, to grow in this nutrient-poor environment. Our data analysis shows that SDH and LDH dehydrogenases, as well as citrate metabolism are activated in urine-like conditions. Moreover, we also observed an increase in the content of CitA, part of a two-component system involved in citrate transport, and the citrate-acetate antiporter CitW. Additionally, the expression of the *α*-ketoglutarate transporter KgtP was also found increased in these conditions. In a similar manner, the LldD lactate dehydrogenase and its related transporter, LldP, were increased in substantial amounts in mAUM, suggesting that lactate is being employed as a metabolite and the enzymatic machinery to transport it and oxidize is being upregulated. Overall, the content of enzymes that carry important roles in the Krebs cycle was increased as an adaptation for the bacteria to growth in urine-like conditions to utilize the substrates present in these conditions.

While the composition of urine varies between individuals, an average composition has been previously reported (Kramer et al., 1972), showing significant amounts of citrate and lactate, at concentrations close to our urine-like formulation (Kramer et al., 1972). Our results suggest that *K. aerogenes* is able to metabolize citrate through the Krebs cycle, which produces succinate, while also being able to use lactate as source of electrons.

Our data show that at least three transporters that mobilize substrates for the Krebs cycle, KtgP, LldP and CitW, have increased expression in cells grown in mAUM. It has been demonstrated that KtgP and LldP are symporters that require protons to translocate their molecular targets (Seol and Shatkin, 1992; Núñez et al., 2002), therefore, a PMF gradient is required for their activity (Zhang et al., 2015). In urine-like media, while NDH-2 functions as the main dehydrogenase in the conditions studied, its activity is unable to contribute to the PMF gradient for these transporters or other processes since it is a non-proton pumping enzyme (Vamshi Krishna and Venkata Mohan, 2019). However, there is an increase in NDH-1 expression in urine-like media, which is able to pump 4 protons into the outer face of the membrane per electron pair (Bekker et al., 2009; Sazanov, 2015), explaining why *K. aerogenes* increases the expression of this dehydrogenase.

We found that the activity of *bd-*I is higher compared to the activity of *bo_3_*, even though the last one pumps more protons per electron (*bo_3_:* H^+^/e^−^ = 2; *bd-*I: H^+^/e^−^ = 1) (Bekker et al., 2009; Borisov et al., 2011b). Interestingly, *K. aerogenes* respiratory chain, composed mainly of NDH-2, SDH and *bd*-I oxidase would appear to pump only two protons per electron pair. Therefore, the respiratory chain that we are reporting is peculiar because the number of protons pumped per electron is limited, making it seemingly inefficient. However, there might be physiological adaptations for this, such as energy expenditure for protein production where the smaller *bd*-I might be preferred, an interest research avenue in the future.

Finally, according to the proteomic data, the expression of the *bo*_3_ terminal oxidases was also significantly increased in mAUM compared to LB, but their activity in both conditions remains the same, which may suggest that these enzymes are starting to be upregulated at this point. On the other hand, *bd*-I structural proteins had a reduced expression, but their biogenesis proteins had a significant fold-increase, which would be an important point of further examination to understand *K. aerogenes* metabolic adaptations. Consequently, our results point toward a metabolic shift in *K. aerogenes* to survive in the nutrient-poor environment of mAUM. This media simulates the human urine, and we demonstrate that this pathogen is well equipped to quickly and efficiently employ the components of this cell-free human-like fluid alone to grow to the stationary phase.

### Concluding remarks

The data presented here describe the respiratory chain of *K. aerogenes*, an opportunistic pathogen of great clinical relevance. We found that the respiratory metabolism of this bacterium differs from other gram-negative species, *K. aerogenes* preferentially uses NDH-2 dehydrogenases to pump electrons into the electron transport chain while also employing SDH and LDH enzymes during growth in urine-like conditions. As shown here, most of the respiratory activity is carried out by a *bd*-I oxidase. However, this bacterium encodes several *bd*-type oxidases, the highest reported to date in a single microorganism, with previously unknown roles. These data are critically important for the development of new drugs which target respiratory metabolism, as bacterial NDH-2 dehydrogenases and *bd*-I oxidoreductases are not present in human cells.

## Data Availability

The original contributions presented in the study are included in the article/Supplementary material, further inquiries can be directed to the corresponding author.
