# Supplementary material for: The respiratory chain of Klebsiella aerogenes in urine-like conditions: critical roles of NDH-2 and bd-terminal oxidases
Source: Front Microbiol. 2024 Nov 6;15:1479714. doi: 10.3389/fmicb.2024.1479714 (PMC11576283; doi:10.3389/fmicb.2024.1479714)
Supplement: Supplementary file 1 [file Table_1.pdf]

The respiratory chain of *Klebsiella aerogenes* in urine-like conditions: critical roles of NDH-2 and *bd*-terminal oxidases

Martín A. González-Montalvo, Jennifer M. Sorescu, Gabriella Baltes, Oscar Juárez and Karina Tuz

List of materials:

Supporting Table 1 -- S-2

Table S1. Queries used for identification of respiratory dehydrogenases and terminal oxidases in *K. aerogenes* 35029

Target	Query (Uniprot ID)	Gene ID (Start-End)
NDH-1	NuoA (F6AED2)	*nuoA* (3,817,621 - 3,818,067)
NQR	NqrA (A0A3S4EW66)	*nqrA* (1,069054 – 1,070,400)
NDH-2 A	Ndh-2 A (F6AED2)	No Hit
NDH-2 B	NDH-2 B (C9Z4Z1)	No Hit
NDH-2 C	NDH-2 C (Q9I0F1)	NDH-2 C (5,018,136 – 5,019,338)
NDH-2 D_1	NDH-2 D_1 (A0A076JG49)	NDH-2 D_2 – 1 (2,043,293 - 2,044,597)<br>NDH-2 D_2 – 2 (1,374,401 – 1,375,705)
NDH-2 D_2	NDH-2 D_2 (A0A0H3FSH0)	NDH-2 D_2 – 1 (2,043,293 - 2,044,597)<br>NDH-2 D_2 – 2 (1,374,401 – 1,375,705)
NDH-2 D_3	NDH-2 D_3 (G2LGF2)	NDH-2 D_2 – 1 (2,043,293 - 2,044,597)<br>NDH-2 D_2 – 2 (1,374,401 – 1,375,705)
NDH-2 D_4	NDH-2 D_4 (B2FIT3)	NDH-2 D_2 – 1 (2,043,293 - 2,044,597)<br>NDH-2 D_2 – 2 (1,374,401 – 1,375,705)
NDH-2 D_5	NDH-2 D_5 (A5K6L8)	NDH-2 D_2 – 1 (2,043,293 - 2,044,597)<br>NDH-2 D_2 – 2 (1,374,401 – 1,375,705)
SDH	SdhA (A0A377RD13)	*sdhA* (1,538,417 – 1,540,183)
LldD	LldD (A0A3S4JB82)	*lldD* (5,123,622 – 5,124,806)
Dld	Dld (A0A3S4I3H8)	*dld* (3,680,207 – 3,681,940)
MQH	Mqo1 (A0A3S4HV18)<br>Mqo2 (A0A3S4G543)<br>Mqo3 (A0A3S4KWQ9)	*mqo1* (2,443,324 – 2,445,021)<br>*mqo2* (3,754,387 – 3,756,021)<br>*mqo1* (2,443,324 – 2,445,021)
PetA	PetA (A0A3L0Y8D6)	No Hit
bc_1	Bc1 (A0A318G4S3)	No Hit
Qcr	QcrA (P0DOV3)	No hit
fbc	FbcC (Q02760)	No Hit
caa_3	CtaD (A0A5E7F276)	*cyoB* (1,233,856 – 1,235,847)
aa_3	QoxB (P34956)	*cyoB*(1,233,856 – 1,235,847)
cbb_3	CcoN (D9IA43)	No Hit
bo_3	CyoA (A0A0F1LA05)	*cyoA* (1,235,867 – 1,236,811)
bd-I	CydA (A0A094XD20)	*cydA*-1 (1,548,196 – 1,549,764)<br>*cydA*-2 (2,551,178 – 2,552,581)<br>*appC*-1 (2,820,548 – 2,821,945)<br>*appC*-2 (2,723,533 – 2,724,942)
bd-II	AppB (A0A9Q7LR64)	*appB*-1 (2,821,945 – 2,822,955)<br>*appB*-2 (2,724,944 – 2,725,954)<br>*cydB*-1 (1,549,780 – 1,550,919)<br>*cydB*-2 (2,552,581 – 2,553,581)

* Gene names are listed in the Gene ID column except for NDH-2, for which the protein name and class are listed.
